# Validation of a novel associative transcriptomics pipeline in *Brassica oleracea*: identifying candidates for vernalisation response

**DOI:** 10.1186/s12864-021-07805-w

**Published:** 2021-07-13

**Authors:** Shannon Woodhouse, Zhesi He, Hugh Woolfenden, Burkhard Steuernagel, Wilfried Haerty, Ian Bancroft, Judith A. Irwin, Richard J. Morris, Rachel Wells

**Affiliations:** 1 Department of Crop Genetics, John Innes Centre, NR47UH Norwich, UK; 2 Department of Biology, University of York, YO105DD Heslington, York, UK; 3 Computational & Systems Biology, John Innes Centre, NR47UH Norwich, UK; 4 Earlham Institute, NR47UH Norwich, UK; 5 School of Biological Sciences, University of East Anglia, NR47TJ Norwich, UK

**Keywords:** Associative Transcriptomics, GWAS, Population Structure, *Brassica oleracea*, Flowering, Vernalisation

## Abstract

**Background:**

Associative transcriptomics has been used extensively in *Brassica napus* to enable the rapid identification of markers correlated with traits of interest. However, within the important vegetable crop species, *Brassica oleracea*, the use of associative transcriptomics has been limited due to a lack of fixed genetic resources and the difficulties in generating material due to self-incompatibility. Within Brassica vegetables, the harvestable product can be vegetative or floral tissues and therefore synchronisation of the floral transition is an important goal for growers and breeders. Vernalisation is known to be a key determinant of the floral transition, yet how different vernalisation treatments influence flowering in *B. oleracea* is not well understood.

**Results:**

Here, we present results from phenotyping a diverse set of 69 *B. oleracea* accessions for heading and flowering traits under different environmental conditions. We developed a new associative transcriptomics pipeline, and inferred and validated a population structure, for the phenotyped accessions. A genome-wide association study identified *miR172D* as a candidate for the vernalisation response. Gene expression marker association identified variation in expression of *BoFLC.*C2 as a further candidate for vernalisation response.

**Conclusions:**

This study describes a new pipeline for performing associative transcriptomics studies in *B. oleracea*. Using flowering time as an example trait, it provides insights into the genetic basis of vernalisation response in *B. oleracea* through associative transcriptomics and confirms its characterisation as a complex G x E trait. Candidate leads were identified in *miR172D* and *BoFLC.C2*. These results could facilitate marker-based breeding efforts to produce *B. oleracea* lines with more synchronous heading dates, potentially leading to improved yields.

**Supplementary Information:**

The online version contains supplementary material available at 10.1186/s12864-021-07805-w.

## Introduction

Ensuring synchronous transiting from the vegetative to the reproductive phase is important for maximising the harvestable produce from brassica vegetables. Many cultivated brassica vegetables arose from their native wild form *B. oleracea* var. *oleracea* [1]. Wild cabbage, *B. oleracea* L., is a cruciferous perennial growing naturally along the coastlines of Western Europe. From this single species, selective breeding efforts have enabled the production of the numerous subspecies we see today. The specialization of a variety of plant organs has given rise to the large diversity seen within the species. Various parts of brassicas are harvested, including leaves (e.g. leafy-kale and cabbage), stems (e.g. kohl-rabi), and inflorescences (broccoli and cauliflower). For all subspecies, the shift from the vegetative to reproductive phase is important and being able to genetically manipulate this transition will aid the development and production of synchronous brassica vegetables.

Determining how both environmental and genotypic variation affect flowering time is important for unravelling the mechanisms behind this transition. For many *B. oleracea* varieties, a period of cold exposure, known as vernalisation, is required for the vegetative-to-floral transition to take place. This requirement for vernalisation, or lack thereof, determines whether the plant is a winter annual, perennial or biennial or whether it is rapid-cycling or a summer annual [2]. As a consequence, the response of the plant to vernalisation provides quantifiable variation that has been exploited by breeders to develop varieties with more synchronous heading. Such variation will be key for future breeding in the face of a changing climate.

Genome-wide association studies (GWAS) are an effective means of identifying candidate genes for target traits from panels of genetically diverse lines [3]. GWAS has been used successfully in numerous plant species including Arabidopsis, maize, rice and Brassica [4–7]. However, its application is reliant on genomic resources which are not always available for complex polyploid crops. Associative transcriptomics uses the sequences of expressed genes (mRNAseq) aligned to a reference to identify and score molecular markers that correlate with trait data. These molecular markers represent variation in gene sequences and expression levels. Therefore, unlike traditional GWAS analysis, associative transcriptomics also enables identification of associations between traits and gene expression levels [4]. Associative transcriptomics is a robust method for identifying significant associations and is being used increasingly to identify molecular markers linked to trait-controlling loci in crops [8–11].

An important factor to account for in association studies is the genetic linkage between loci. If the frequency of association between the different alleles of a locus is higher or lower than what would be expected if the loci were independent and randomly assorted, then the loci are said to be in linkage disequilibrium (LD) [12]. LD will vary across the genome and across chromosomes and it is important to account for this in GWAS analyses. This variation in LD is due to many factors, including selection, mutation rate and genetic drift. Strong selection or admixture within a population will increase LD. Accounting for the correct population structure reduces the risk of detecting spurious associations within GWAS analyses. The population structure can be determined from unlinked markers [13].

Here, we develop and validate an associative transcriptomics pipeline for *B. oleracea.* A specific population structure consisting of unlinked markers was generated using SNP data from 69 lines of genetically fixed *B. oleracea* from the Diversity Fixed Foundation Set [14]. The pipeline was successfully used for the identification of candidate leads involved in vernalisation response, identifying a strong candidate in *miR172D*.

## Results

### Exposure to different environmental conditions identifies vernalisation requirements across the phenotyped accessions

**Fig. 1:**
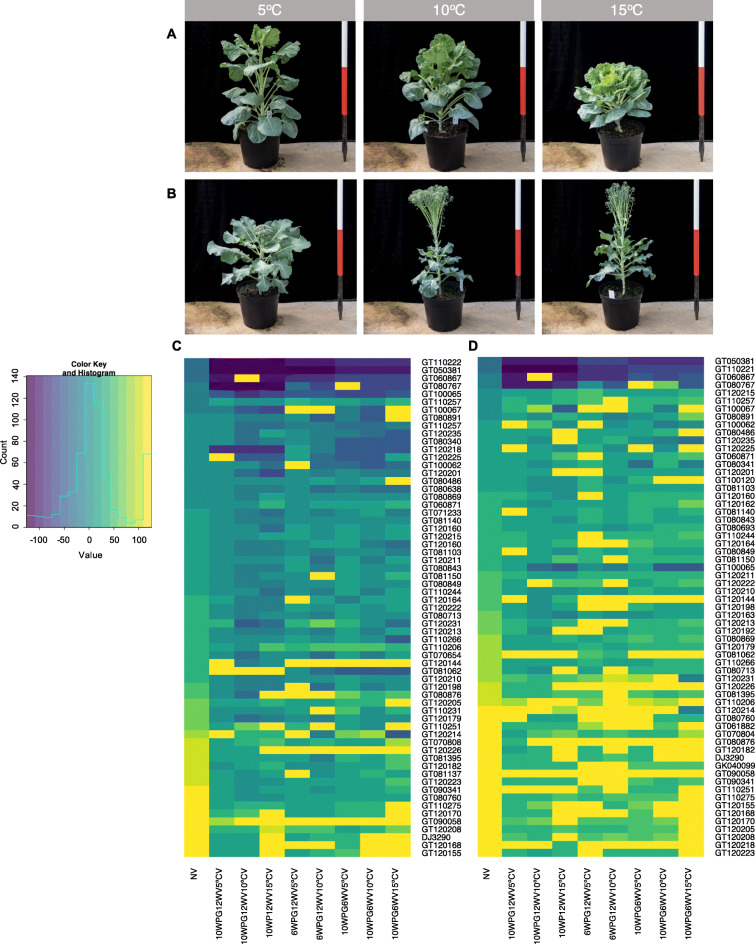
Flowering time traits exhibit a varied response to different environmental conditions within the population. Examples of opposing phenotypic response to different vernalisation temperatures can be observed in (**A**) Brussels Sprout, Cavolo Di Bruxelles Precoce (GT120168) and (**B**) Broccoli, Mar DH (GT110244). Variation across the population for (**C**) DTB post vernalisation per treatment, per line. (**D**) DTF post vernalisation per treatment, per line. Day 0 represents the end of vernalisation, negative values represent heading or flowering during the pre-growth or vernalisation

We selected a subset of 69 *B. oleracea* lines, diverse in both eco-geographic origin and crop type, from the *B. oleracea* Diversity Fixed Foundation Set [14]. We used these accessions to evaluate the importance of vernalisation parameters by quantifying flowering time under different conditions (vernalisation start, duration and temperature). Two key developmental stages were monitored: ‘days to buds visible’ (DTB) and ‘days to first flower’ (DTF). The variation in flowering time across the different treatments and between the different lines is shown in Fig. 1. The different vernalisation start times demonstrate that exposure to the longer, ten-week pre-vernalisation growth period (10WPG) typically results in earlier flowering, compared to the shorter, six-week pre-growth period (6WPG). The mean DTB for 6WPG was 21.0 days (SD = 51.6), compared to 5.8 days (SD = 49.9) for the 10WPG (Wilcoxon Test, W = 17,958, *P* = 0.004). Similarly, we found a significant difference in the time taken to reach DTF between the two treatment groups, with a mean of 57.9 days (SD = 55.5) following the 6WPG, in comparison to 35.9 days (SD = 53.1) following the 10WPG (Wilcoxon Test, W = 17,471, *P* = 2.96e-05).

Changes in vernalisation duration led to a significant difference in DTB, but not in DTF. Following the six-week vernalisation (6WV), the mean DTB was 9.5 days (SD = 44.5) compared to 5.8 days (SD = 46.8) after exposure to twelve-weeks of vernalisation (12WV) (Wilcoxon Test, W = 19,532, *P* = 0.002). This difference was coupled with more synchronous heading between lines following the 12WV period. The impact of vernalisation duration on DTB varied across the population, reflecting the numerous factors that can affect DTB depending on crop type, such as stem elongation and developmental arrest.

Of the three parameters we investigated, vernalisation temperature resulted in the most pronounced phenotypic differences. The 5ºC vernalisation (5 ºCV) resulted in the largest DTB (slowest overall bud development), whereas the 10ºC vernalisation (10 ºCV) treatment resulted in the largest DTF. The distribution between heading dates was distinctly different between the temperatures. Higher vernalisation temperatures resulted in larger the variation in DTB and DTF. The more synchronous heading and flowering for the 5ºCV treatment suggests that this temperature was able to saturate the vernalisation requirement for a large proportion of the lines. After exposure to the warmer temperatures, the variation in DTB and DTF were greatly increased (Additional File 1), indicating that the cooler vernalisation temperature aided faster transitioning in some lines, but delayed the development of others. This is consistent with differences in *B. oleracea* crop types, for example Brussels Sprouts are known to have a strong vernalisation requirement, whereas Summer Cauliflower have been bred to produce curd rapidly without the need for cold exposure [15, 16].

The effect of vernalisation temperature on the floral transition is demonstrated clearly between the Broccoli Mar DH and the Brussel Sprout Cavolo Di Bruxelles Precoce (Fig. 1 A), with polar responses to vernalisation temperature. Mar DH transitioned fastest under the 15 ºC vernalisation (15 ºCV) treatment, whereas Cavolo Di Bruxelles Precoce transitioned faster under the 5 ºCV treatment. Faster transitions at higher vernalisation temperatures as in the case of Mar DH, however, can lead to undesirable phenotypes from a grower’s perspective (Fig. 1B).

### Unlinked markers are required to generate a representative population structure

GWAS requires trait, SNP and population data. The correct population structure is important for ensuring that associations are with the trait of interest rather than identified on account of relatedness within the population, in particular for panels of only one species. To generate a representative population structure, it is necessary to ensure the SNPs used are unlinked [13]. However, different criteria have been used to select these SNPs [6, 17–19]. To evaluate the impact of SNP selection criteria, we generated two population structures and investigated their suitability for representing the panel.

Using all markers with a minor allele frequency (MAF) larger than 0.05 [4, 20, 21], reduced the total number of SNPs from 110,555 to 36,631. Calculation of ΔK showed a maximum value of K = 2, although a further peak in ΔK was observed at K *=* 5 (Additional File 6 A), thus identifying substructure within the population. ΔK frequently identifies K = 2 as the top level of hierarchical structure, even when more subpopulations are present [21, 22]. Subsequent phylogenetic analysis (Additional File 7 A, 7B) identified clusters representing these sub populations. Therefore, to account for substructure within the population, the value of K = 5 was used for further analysis [22, 23]. A second population structure was generated using stricter parameters, requiring the markers be biallelic, MAF > 0.05, one per gene and at least 500 bp apart. A total of 664 SNPs met these requirements, resulting in the identification of four subpopulation clusters (Additional File 4).

**Fig. 2:**
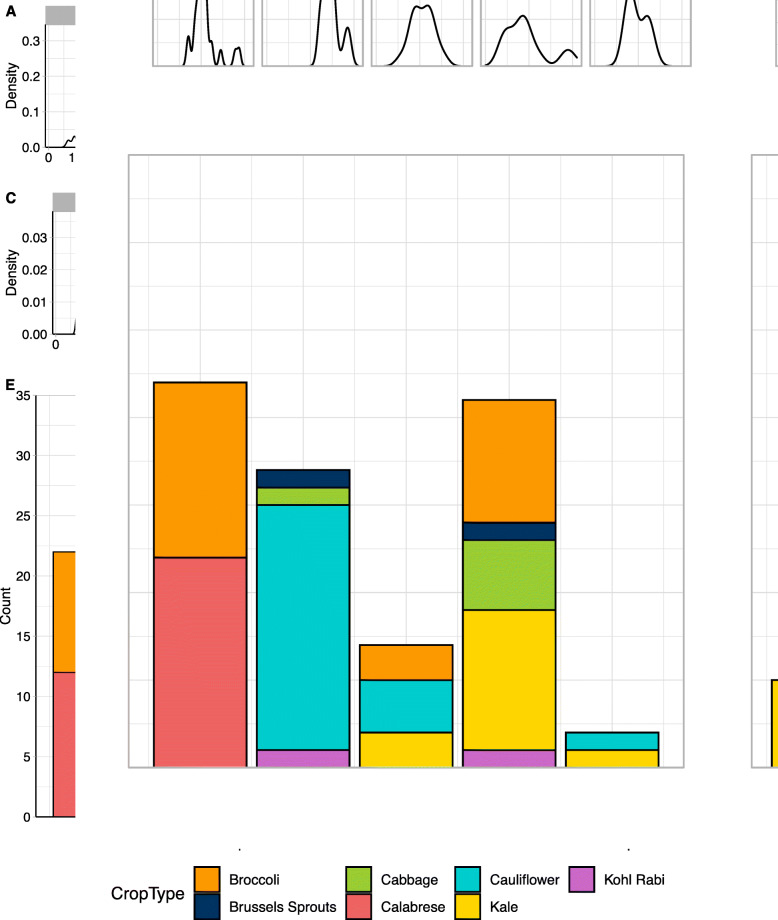
The choice of SNP pruning rules can significantly change the inferred population structure. Density plots representing (**A**) DTB, **C**) DTF for the accessions within the five subpopulation clusters. Density plots representing (**B**) DTB, D) DTF for the accessions within the four subpopulation clusters. E) Population structure generated from SNPs with MAF > 0.05 F) Population structure generated from more stringent SNP pruning (Biallelic only, MAF > 0.05, > 500-bp apart, one per gene)

We assessed the two population structures based on crop type and phenotypic data. Using K = 5, generated using the less stringent parameters, (Fig. 2 A, 2 C, 2E) cluster one contained only broccoli and calabrese, both members of the same subspecies var. *italica* [24, 25], whereas cluster two mainly comprised cauliflower, subspecies var. *botrytis*. Late flowering accessions were included in both clusters. Interestingly, this population structure grouped the rapid cycling and late flowering kales together with a spread of accessions from other crop types, in cluster four. The remaining two clusters were small by comparison: cluster three comprised of seven accessions, a mixture of broccoli, cauliflower and kale; cluster five consisted of just two lines, one kale and one cauliflower.

The four clusters identified using more stringent SNP selection criteria contained all of the rapid cycling kales in cluster one, characterised by their early heading and flowering phenotypes (Fig. 2B and D F). This was identified as a clear subgroup within the phylogenetic tree (Additional File 7 C). Cluster two was mainly broccoli and calabrese, whilst cluster three consisted largely of the earlier flowering cauliflowers. Cluster four contained the late flowering individuals from all crop types within the population, hence the larger variation in heading and flowering for this cluster.

Comparison of the clustering of accessions between the two population structures demonstrated the more stringent SNP criteria gave rise to a population structure in which individuals were grouped with other accessions that would be expected to be genetically similar based on knowledge of crop type and flowering phenotype. Consequently, this population structure was applied in subsequent GWAS analyses.

To gauge the extent of linkage disequilibrium we calculated the mean pairwise squared allele-frequency correlation (*r*^*2*^) for mapped markers. A linkage disequilibrium window of 50 (providing > 3 million pairwise values of *r*^*2*^) resulted in a mean pairwise *r*^*2*^ of 0.0979, confirming a low overall level of linkage disequilibrium in *B. oleracea*.

### Associative transcriptomics identifies *miR172D* as a candidate for controlling vernalisation response

SNP associations were compared to the physical positions of orthologues of genes known to be involved in the floral transition in Arabidopsis. A total of 43 flowering time related traits (Additional File 2) were analysed using this pipeline, including DTB and DTF for each treatment. A total of 111 significant SNPs were identified, *P* < 0.05, six of which demonstrated clear association peaks and were investigated further (Table 1).

**Table 1:** Significant SNP associations with vernalisation response in diverse *B. oleracea* accessions, detected across the genome (FDR < 0.05), including model information

Marker information			Association information					Model information	
Marker	Chromosome	Alleles	-Log10(p)	Marker R2	Traits	Arabidopsis ID	Orthologue	Model	Population structure correction
Bo6g103650.1:2010:T	C06	C/T/Y	6.4017787	0.39231	6P 12 V 10 °C DTB	AT1G67140.3	*SWEETIE*	GLM	Q-Matrix
Bo9g179000.1:2589:G	C09	G/T/K	6.4077566	0.39662	6P 12 V 10 °C DTB	AT5G04240.1	*ELF6*	GLM	Q-Matrix
Bo1g011280.1:786:A	C01	A/T/W	6.0844894	0.44220	10P 12 V 5 °C DTF	AT4G31490.1	Coatomer, beta subunit	GLM	Q-Matrix
Bo7g026810.1:124:G	C07	A/G/R	4.7781947	0.36476	6P 12 V 10 °C DTF	AT2G05790.1	O-Glycosyl hydrolases family 17 protein	GLM	PCA
Bo7g104810.1:204:T	C07	A/T/W	5.9788107	0.41678	10P 6 V 15–5 °C DTB	AT3G55512	*mir172D*	GLM	Q-Matrix
Bo2g009460.1:894:T	C02	C/T	7.6880767	0.40565	10P 6 V 5 °C DTF - DTB	AT5G10140.4	*FLC.C2*	GLM	Q-Matrix

**Fig. 3:**
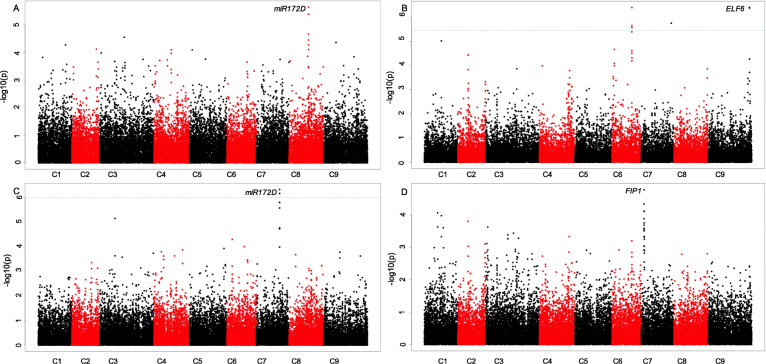
The developed pipeline identifies associations with flowering traits. Distribution of mapped markers associating with (**A**) Number of DTF under non-vernalising conditions (**B**) DTB after a six-week pre-growth, twelve weeks vernalisation 10 ºC (**C**) The difference in DTB between six and twelve weeks of vernalisation at 15 ºC, after exposure to a ten-week pre-growth (**D**) The DTF after exposure to six-week pre-growth, twelve weeks vernalisation 10 ºC. Sixty-nine accessions of *B. oleracea* were phenotyped for DTB and DTF and marker associations were calculated using a generalized linear model, implemented in TASSEL to incorporate population structure. Log_10_ (P values) were plotted against the nine *B. oleracea* chromosomes in SNP order. Blue line FDR threshold, P < 0.05, FDR threshold was not met for **A**) and **D**)

We first sought to identify genetic associations with the trait data for the non-vernalised experiment. Whilst no significant association peaks were identified for DTB, a single marker association at Bo8g089990.1:453:T was identified (P = 2.29E-06) for DTF under non-vernalising conditions. This marker was within a region demonstrating good synteny to Arabidopsis, despite there being a number of unannotated gene models present. Conservation between Arabidopsis and *B. oleracea* suggests that this region contains an orthologue of *microRNA172D*, AT3G55512, which has been linked to the floral transition in *A. thaliana* [26, 27] (Fig. 3 A). Furthermore, the difference in DTB between 10WPG6WV5 ºCV and 10WPG12WV15 ºCV, identified a significant association on C07 at Bo7g104810.1:204:T (FDR, P < 0.05). This association was in the vicinity of a second orthologue of *miR172D* (Fig. 3 C).

We then analysed the association with traits relating to the timing of vernalisation. No significant associations were identified for traits after 6WPG12WV5 ºCV. However, a strong association was identified on C07 at the marker Bo7g026810.1:124:G, for DTF for 6WPG12WV10 ºCV. Synteny with Arabidopsis suggests that an orthologue of *FRI INTERACTING PROTEIN 1*, (*FIP1*), AT2G06005.1 (Fig. 3D) is present within this region. Within Arabidopsis it has been demonstrated that FIP1 interacts with FRIGIDA (FRI) [28] which is a major source of natural variation in flowering time in Arabidopsis and has been shown to be important in determining vernalisation requirement. Additionally, significant associations (FDR, P < 0.05), were found for DTB for 6WPG12WV10 ºCV. An association was identified at Bo9g179000.1:2589:G, which is in the vicinity of an orthologue of *EARLY FLOWERING 6* (*ELF6*), AT5G04240.1 (Fig. 3B), a nuclear targeted protein able to affect flowering time irrespective of *FLC.*

**Fig. 4:**
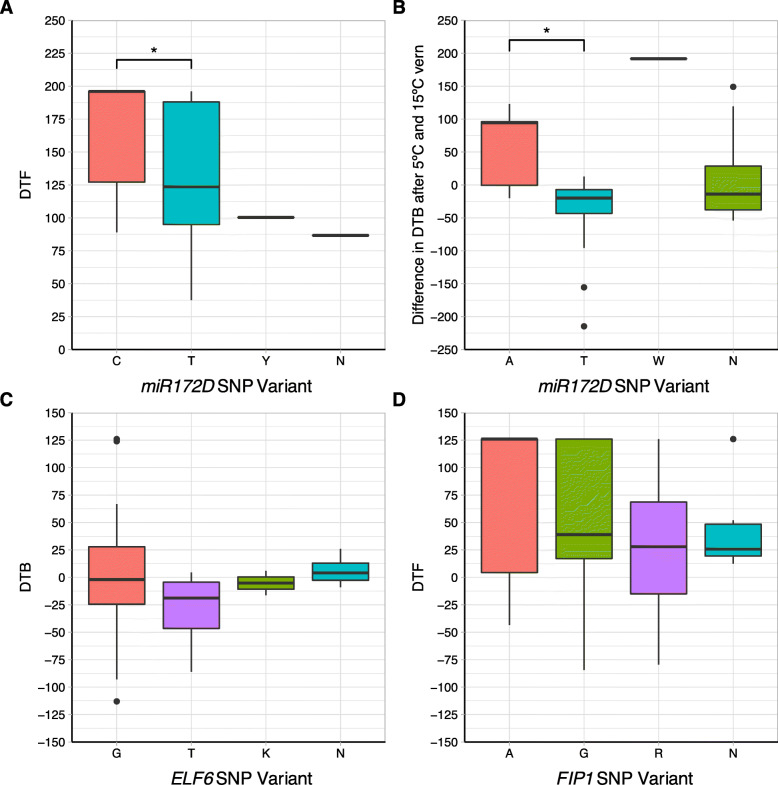
A significant phenotypic difference was found for individuals exhibiting SNP variants for the associations pointing to *miR172D* as a candidate. Boxplots represent the trait data, DTB or DTF for each of the significant markers alongside the different alleles present across the population for each marker. The box represents interquartile range, outliers are represented by black dots

The differences in flowering phenotype between the SNP variants for the four strongest associations were analysed (Fig. 4). There were significant differences in the traits associated with *miR172D* (DTF with no vernalisation and the difference in DTB for plants grown under 5 ºCV and 15 ºCV) for different alleles (Fig. 4 A and B). For Bo7g104810.1:204:T (difference in DTB after exposure to 5 ºCV and 15 ºCV), five individuals, four broccoli and one cauliflower, contained the A variant. The alternate variant, a T allele, and was present in 50 individuals. Conversely, Bo8g089990.1:453:T (DTF with no vernalisation) had 11 individuals with a C allele at this locus, whilst 51 had a T allele. Interestingly, individuals with the C allele were present in every crop type.

### Associative transcriptomics identifies *Bo****FLC.C2*** as a candidate gene involved in vernalisation requirement in ***B. oleracea***

**Fig. 5:**
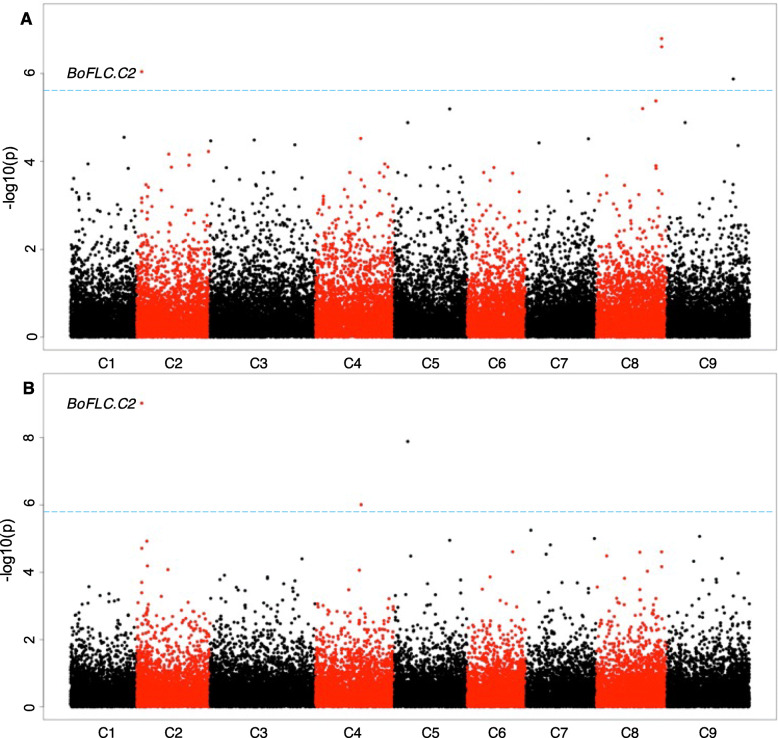
GEM analysis identifies *FLC* expression on chromosome C2 as a candidate for flowering traits under non-vernalising conditions. Distribution of gene expression markers associating with (**A**) DTB after exposure to non-vernalising conditions (**B**) DTF after exposure to non-vernalising conditions. Log_10_ (*P* values) were plotted against the nine *B. oleracea* chromosomes in SNP order. Blue line FDR threshold, *P* < 0.05

**Fig. 6:**
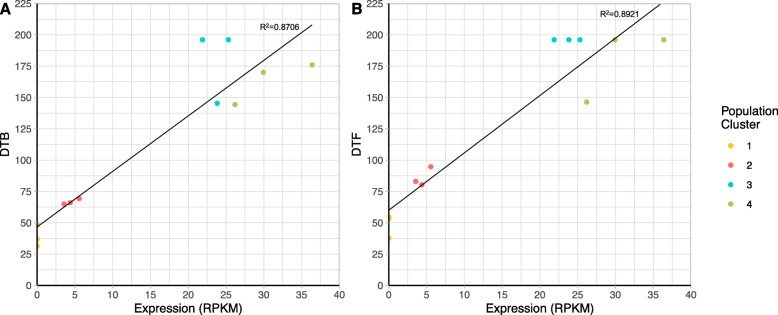
A strong positive correlation can be seen between lines at the phenotypic extremes and their *BoFLC.C2* expression levels. Colours represent the subpopulation of each line, as determined by population structure analysis

An advantage of performing associative transcriptomics as opposed to GWAS, is the additional ability to identify associations between gene expression and the trait of interest. GWAS analysis identified an association of the difference between DTB and DTF with a 10WPG6WV5 ºCV with a candidate marker in the well characterized flowering time gene, *BoFLC.C2* (Table 1). Using gene expression marker (GEM) analysis, *BoFLC.C2* expression was also identified as being significantly associated with both the DTB and DTF under non-vernalising conditions (Fig. 5). *BoFLC.C2* exhibited both low and high expression within the population. As expected, all five rapid cycling accessions demonstrated no *BoFLC.C2* expression. Recently, a Brassica consortium developed targeted sequence capture for a set of relevant genes, including *FLC*. DNA from four of the five rapid cycling accessions had been enriched with that capture library and sequenced. Lacking a reference sequence for *B. oleracea* that contains *BoFLC.C2*, we used *B. napus* (cv. Darmor) [29] as a reference to map the captured sequence data from the four rapid cycling accessions to. Comparison of *B. oleracea* transcript data [30] to this Darmor genome reference revealed a 99.54 % identity in coding sequence, allowing Darmor to be used as a surrogate reference. Indeed, we found that *BoFLC.C2* was absent from all four rapid cycling accessions, GT050381, GT080767, GT100067 and GT110222, revealed by a lack of read mapping (Additional File 10). *BoFLC.C2* is known to be involved in vernalisation response [30] and rapid cycling varieties do not require a period of vernalisation in order to transition to the floral state. As a control, we investigated mapping for 49 non-rapid cycling accessions where we expect *BoFLC.C2* to be present. For all 49 we found the expected read mapping evidence, confirming that use of the polyploid *B. napus* reference is appropriate (Additional File 10). The control of flowering is a complex, multigenic trait, therefore we would not expect a single locus to explain all variation across the entire dataset. Indeed, only a weak positive correlation (DTB *R*^2^ = 0.024, DTF *R*^2^ = 0.036) between flowering phenotype and *BoFLC.C2* expression was identified. A strong positive correlation (DTB *R*^2^ = 0.871, DTF *R*^2^ = 0.891) was found for the phenotypic extremes (rapid cycling lines with no expression and the late flowering lines with high levels of *BoFLC.C2*), Fig. 6, confirming a role for *BoFLC.C2*.

## Discussion

Determining which genes underly phenotypic traits is a key step for crop improvement. A powerful approach for identifying candidates is associative transcriptomics, which has been implemented for several crops. However, for the important vegetable crop *B. oleracea*, no such pipeline has been published to date. Here we present a validated associative transcriptomics pipeline for *B. oleracea* and use it to identify gene candidates for vernalisation.

To reduce the risk of false positives, we developed stringent criteria to identify unlinked markers for the determination of the population structure. The population structure was validated using crop type and phenotypic information on heading and flowering, this example was chosen as producing synchronous *B. oleracea* vegetables is a key goal for growers and breeders. Quantifying vernalisation responses for different varieties is an important step towards this goal, providing a foundation for targeted breeding.

Phenotyping for both DTB and DTF under different environmental conditions revealed a varied response within the population and identified some general trends. Altering the timing of vernalisation demonstrated that a shorter growth period prior to the exposure to cold extended the time taken to reach DTB and DTF. This could be attributed to the presence of a juvenile phase in many of the lines, which has been widely documented in *B. oleracea* [14, 31, 32]. A juvenile plant is described as being unable to respond to floral inductive cues. The fact that many lines were able to flower much faster following longer pre-vernalisation growth, suggests they had reached the adult vegetative phase and were receptive to cold as a floral inductive cue. Further experimental work would be needed to test this hypothesis.

Increasing vernalisation length and reducing vernalisation temperature resulted, on average, in faster and more synchronous heading and flowering. This was a predicted outcome, as current knowledge suggests that increased vernalisation duration and cooler vernalisation temperatures would saturate the vernalisation requirement of a larger proportion of accessions.

Using our validated population structure with associative mapping, we identified candidates orthologous to known Arabidopsis floral regulators, including *miR172D*. In Arabidopsis, the miR172 family post-transcriptionally supress a number of *APETALA1-*like genes, including *TARGET OF EAT1, 2* and *3*, which in turn aids the promotion of floral induction [27, 33–35]. Furthermore, the SNP variant data for both associations implicating miR172D, exhibit significant phenotypic differences. Two orthologues of Arabidopsis *miR172D* have been identified in *B. oleracea* [36] but their functional roles have yet to be determined.

GWAS analysis identified a significant association with *BoFLC.C2* and the difference in DTB and DTF following a ten-week pre-growth period, with six weeks of vernalisation at 5 ºC. *BoFLC.C*2 is a well characterized flowering time gene [30] and the ability of the GWAS pipeline to identify a known candidate gives confidence in the method. Furthermore, GEM analysis identified *BoFLC.C2* expression as being significantly associated with both DTB and DTF under non-vernalising conditions, which can be attributed to the extreme phenotypes within the population (Fig. 6). No *BoFLC.C2* expression was detected in five lines. A loss-of-function mutation at *BoFLC.C2* in cauliflower has been associated with an early flowering phenotype [37], indicating that *BoFLC.C2* has an equivalent role in cauliflower to *FLC* in Arabidopsis. Four of the five lines for which *BoFLC.C2* expression could not be detected did not have the *BoFLC.C2* paralogue according to the bait capture sequencing data. These four lines were all kales and demonstrated an early flowering phenotype, suggesting that *BoFLC.C2* has a similar role to *AtFLC* in kales, and potentially across *B. oleracea*. Although DTB and DTF were highly correlated with *BoFLC.C2* expression under non-vernalising conditions for the phenotypic extremes, for the whole population the correlation was low. This is to be expected as *BoFLC.C2* is just one of many genes that we expect to be involved in the floral transition within *B. oleracea* and therefore is unlikely to account for all the observed variation.

The expression data used for the GEM analysis was generated from leaf tissue at one timepoint. As a consequence, any genes which are not expressed in the leaf at this time will not be identified in this analysis. Use of transcriptome data from other tissues in addition to the leaf data could identify a greater number of associations.

## Conclusions

Identifying genes underlying phenotypic traits in *B. oleracea* is an important step for the improvement of brassica vegetables. Here, we generate and validate a novel pipeline for associative transcriptomics analysis in *B. oleracea* and show that this pipeline is effective in identifying genetic regulators of complex traits, such as flowering time, demonstrating this approach can be utilised for other traits of agronomic importance, such as germination, quality traits and disease resistance. GWAS analysis identified *miR172D* as a candidate for vernalisation response, whilst GWAS and GEM analysis identified a significant marker at *BoFLC.C2*, an important gene in the vernalisation pathway of *B. oleracea*. Our results provide insight into the genetic control of flowering in *B. oleracea*, and candidates which could provide a foundation for future breeding strategies.

## Methods

### Plant Materials and Growth Conditions

A subset of 69 lines fixed as doubled haploids (DH) or at S4 and above were chosen from the *Brassica oleracea* Diversity Fixed Foundation Set [14] (Additional File 1) comprising accessions from seven different *B. oleracea* crop types; cabbage, cauliflower, calabrese, broccoli, kohl rabi, kale and Brussels sprout. Plants were grown in cereals mix (40 % Medium Grade Peat, 40 % Sterilised Soil, 20 % Horticultural Grit, 1.3 kg/m³ PG Mix 14-16-18 + Te Base Fertiliser, 1 kg/m³ Osmocote Mini 16-8-11 2 mg + Te 0.02 % B, Wetting Agent, 3 kg/m³ Maglime, 300 g/m³ Exemptor) and given a pre-growth period of either six or ten weeks in a glasshouse under natural light supplemented with LED lighting (16 h daylength 21/18 °C day/night). At the end of the pre-growth period, three plants of each line for each treatments were transferred to Conviron controlled environment rooms for six or twelve weeks vernalisation at 5, 10 or 15 ºC (16 h daylength LED, 60 % humidity). Following vernalisation, plants were re-potted into 2 L pots and placed into a polytunnel under natural light using a randomised block design. All plants came out of vernalisation and into the polytunnel on the same day due to staggered sowing to control for post-vernalisation environmental conditions. Three replicates of each line were grown without vernalisation as a non-vernalised control group. The plants were scored at buds visible (DTB) and upon opening of first flower (DTF) [38]. A summary of pre-growth and vernalisation conditions and traits analysed is given in Additional File 2.

### SNP Calling

The growth conditions, sampling of plant material, RNA extraction and transcriptome sequencing was carried out as described by He et al. [39]. The RNA-seq data from each accession were mapped on to CDS models from the *Brassica oleracea* pangenome [40] as reference sequences, using Maq v0.7.1 [41]. SNPs were called by the meta-analysis of alignments as described in Bancroft et al. [42]. SNP positions were excluded if they had a read depth < 10, a base call quality < Q20, missing data > 0.25, and > 3 alleles. This resulted in a SNP file containing 110,555 SNPS, and 65,017 unigene sequences with associated RPKM values.

### Population Structure and GWAS analyses

Population structure was generated using both relaxed (all markers with a minor allele frequency (MAF) > 0.05) and stringent criteria using STRUCTURE [43] (burn-in10000, MCMC 10,000, 10 iterations). For the stringent criteria, SNPs were required to be biallelic, with a minor allele frequency (MAF) > 0.05 and a minimum distance of 500-bp between markers. STRUCTURE HARVESTER [44] was used to determine the optimal *K* value. The Q matrix used in GWAS analysis was calculated using CLUMPP [45].

TASSEL [46] version 5.0 was used to select the most appropriate model for each trait based on QQ plots. Generalised linear models (GLM), with correction for population structure using the Q matrix or PCA (5 PCs) were used to look for associations. For GWAS analysis only SNP markers with an allele frequency > 0.05 were used. To gauge the extent of linkage disequilibrium, the mean pairwise *r*^*2*^ was calculated using the SlidingWindow function within TASSEL, with a linkage disequilibrium window of 50. TASSEL was used to construct phylogenetic trees, using the Neighbour Joining method and all SNPs with MAF > 0.05. Trees were graphed in R using the package ggtree [47].

Gene expression marker (GEM) associations were calculated by an in-house script in R Version 3.6.3 using a fixed effect linear model with RPKM values, excluding markers with an average expression below 0.5 RPKM. Linear regression was performed using RPKM as a predictor value to predict a quantitative outcome of the trait value. Both SNP and GEM outputs were plotted as Manhattan Plots created using an in-house R script. All scripts are available at https://github.com/JIC-CSB/Boleracea-AssociativeTranscriptomics. Statistical significance for both GWAS and GEM association was determined by the false discovery rate (FDR) [48] calculated using the QValue package [49] in R.

### DNA Extraction

Genomic DNA of accessions used in bait capture sequencing was prepared from young leaf tissue of plants grown in a glasshouse (16 h LED supplementary light, 21/18 °C day/night). Light was excluded for 48 h prior to harvesting. Nuclei were extracted from ~ 3 g of tissue prior to CTAB based DNA extraction. Extracts were treated with RNase T1, RNaseA and Proteinase K to remove RNA and protein contamination, respectively. DNA was resuspended in 50 µl dH_2_O and checked for quality. DNA was quantified by and stored at -20 °C.

### Targeted Sequence Enrichment analysis

A bait library for targeted sequence enrichment for a specific subset of genes was developed and synthesized with Arbor Biosciences (https://arborbiosci.com/). Samples were 4 plexed and run on the NovaSeq S4, PE150, 1Gbp/library. Reads from individual accessions were mapped to the reference sequence of *B. napus* cv. Darmor-*bzh* [29] using BWA [50] version 0.7.17-r1188 using aln/sampe and standard parameters. Mapped reads were sorted and indexed using SAMTOOLS [51] version 1.10 sort and index, and subsequently visualized with Integrative Genomics Viewer (IGV) [52].

## Supplementary Information

**Additional file 1: **Details of phenotyped panel, with associated crop type, subspecies and EcoTILLING information.

**Additional file 2: **List of conditions and traits run through the associative transcriptomics pipeline.

**Additional file 3: **Phenotyping results, mean DTB and DTF under all treatments tested.

**Additional file 4: **Analysis of the smaller SNP data set with the Bayesian clustering algorithms implemented in the program STRUCTURE, identified four population clusters.

**Additional file 5: **Increased synchrony in DTB and DTF was observed as vernalisation temperature was reduced. Histograms representing the distribution of DTB and DTF post-vernalisation across the population after exposure to vernalization at 5, 10 or 15 oC. Individuals that did not flower have been removed from this plot.

**Additional file 6: **ΔK based on rate of change of LnP, Maxima indicates the ΔK that best explains the population structure. Plots produced using STRUCTURE Harvester output. A) ΔK values for biallelic SNPs, MAF > 0.05, one SNP per gene, >500kb apart, K = 4. B) ΔK values calculated for SNPs with MAF > 0.05, K = 5.

**Additional file 7: **Phylogenetic trees, generated in TASSEL using the Neighbour Joining method, to demonstrate the substructure present within the phenotyped panel. A) K = 2, the highest level of structure seen within the population following analysis with the relaxed SNP set, B) K = 5, the substructure present within the population following analysis with the relaxed SNP set. C) K = 4, the result following population structure analysis on the stringent SNP set.

**Additional file 8: **Quantile-Quantile Plots for SNP associations with A) the DTF under NV conditions. GLM, with Q matrix correction for population structure B) the DTB after six-week pre-growth and 10ºC vernalisation for twelve-weeks. GLM, with Q matrix correction for population structure C) The difference in DTB following 5 ºC and 15 ºC vernalisation for six-weeks, after exposure to a ten-week pre-growth. GLM with Q matrix correction for population structure D) The DTF after exposure to six-week pre-growth, twelve weeks vernalisation 10 ºC. GLM with PCA correction for population structure.

**Additional file 9: **Linkage disequilibrium decay. A) Bo8g089990.1:453:T, miR172D candidate. B) Bo9g179000.1:2589:G, ELF6 candidate. C) Bo7g026810.1:124:G, FIP1 candidate. D) Bo7g104810.1:204:T, miR172D candidate.

**Additional file 10: **Mapping BoFLC.C2 using Darmor-bzh as a reference. Four rapid cycling accessions and three representative accessions for the rest of the population.

## Data Availability

Sequence data from this article can be found in the SRA data library under accession number PRJNA309368, https://www.ncbi.nlm.nih.gov/bioproject/PRJNA309368. The R scripts used to carry out GEM analysis and to generate the corresponding Manhattan plots for both GEM and GWAS analysis are available on GitHub in the JIC_CSB/Boleracea-AssociativeTranscriptomics repository, DOI 10.5281/zenodo.4529809 [53]. Raw data for targeted sequence capture experiments has been deposited at EBI, under study number PRJEB43076, https://www.ebi.ac.uk/ena/browser/view/PRJEB43076, and the bait library is available at DOI 10.5281/zenodo.4473283 [54].
