# Supplementary figures and images for: Validation of a novel associative transcriptomics pipeline in Brassica oleracea: identifying candidates for vernalisation response
Source: BMC Genomics. 2021 Jul 13;22:539. doi: 10.1186/s12864-021-07805-w (PMC8278714; doi:10.1186/s12864-021-07805-w)

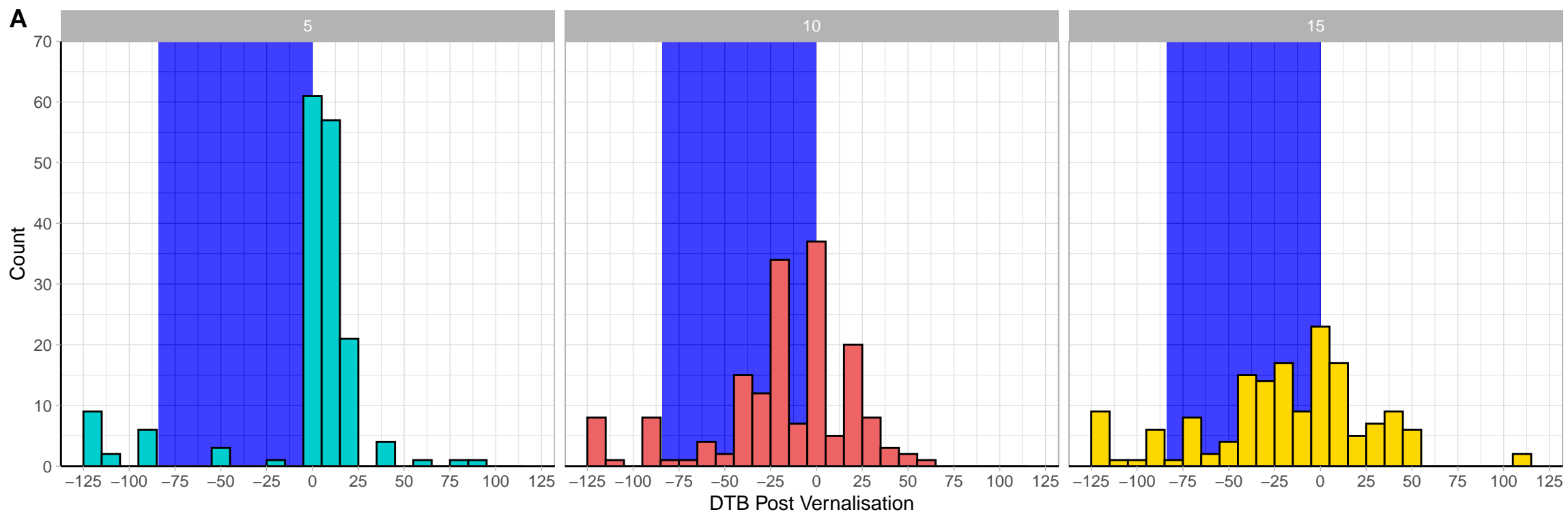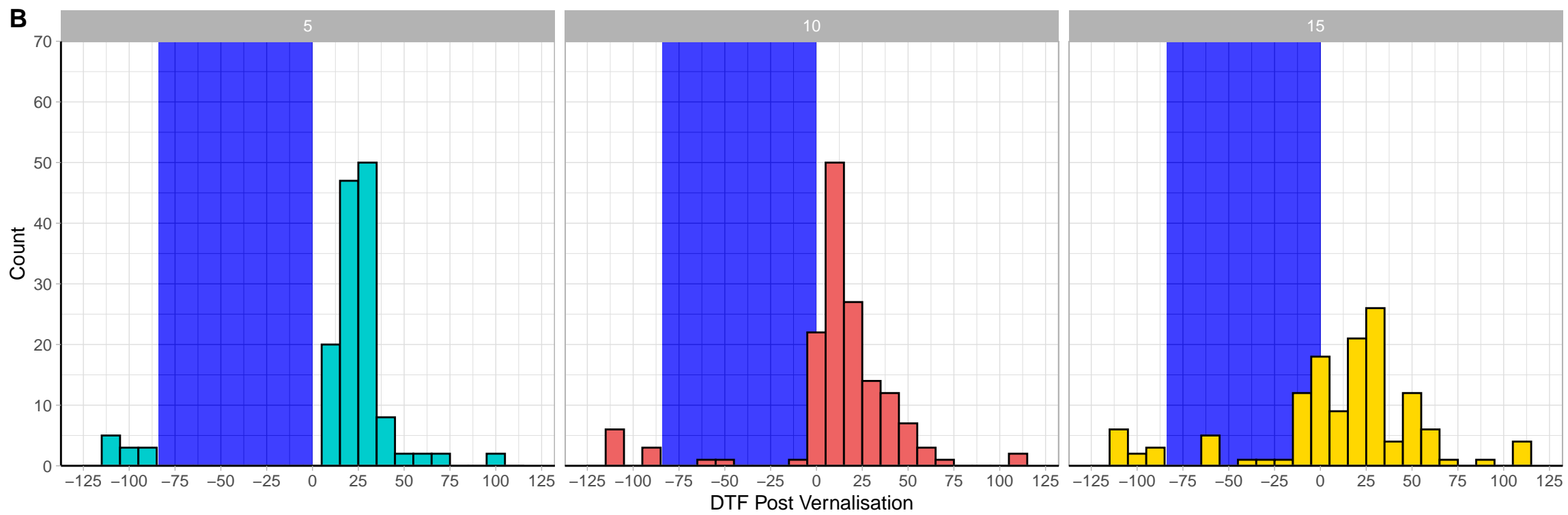

Supplement: Supplementary file 5 — Additional file 5: Increased synchrony in DTB and DTF was observed as vernalisation temperature was reduced. Histograms representing the distribution of DTB and DTF post-vernalisation across the population after exposure to vernalization at 5, 10 or 15 oC. Individuals that did not flower have been removed from this plot. [file 12864_2021_7805_MOESM5_ESM.pdf]

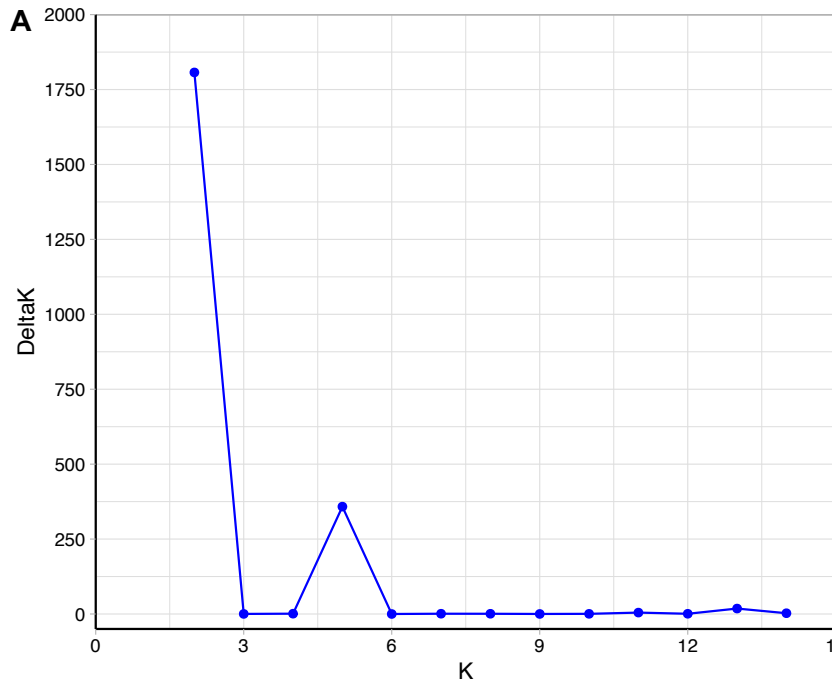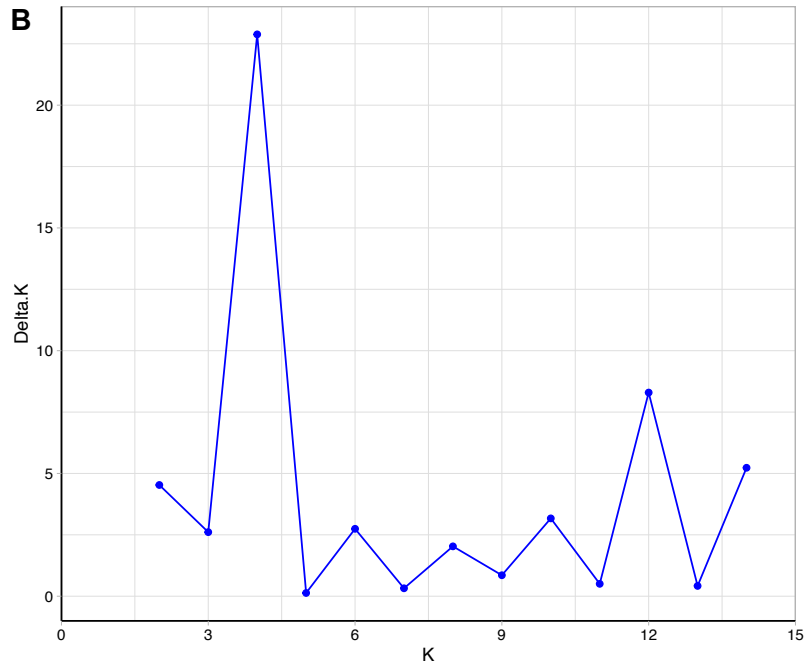

Supplement: Supplementary file 6 — Additional file 6: ΔK based on rate of change of LnP, Maxima indicates the ΔK that best explains the population structure. Plots produced using STRUCTURE Harvester output. A) ΔK values for biallelic SNPs, MAF > 0.05, one SNP per gene, >500kb apart, K = 4. B) ΔK values calculated for SNPs with MAF > 0.05, K = 5. [file 12864_2021_7805_MOESM6_ESM.pdf]

**A**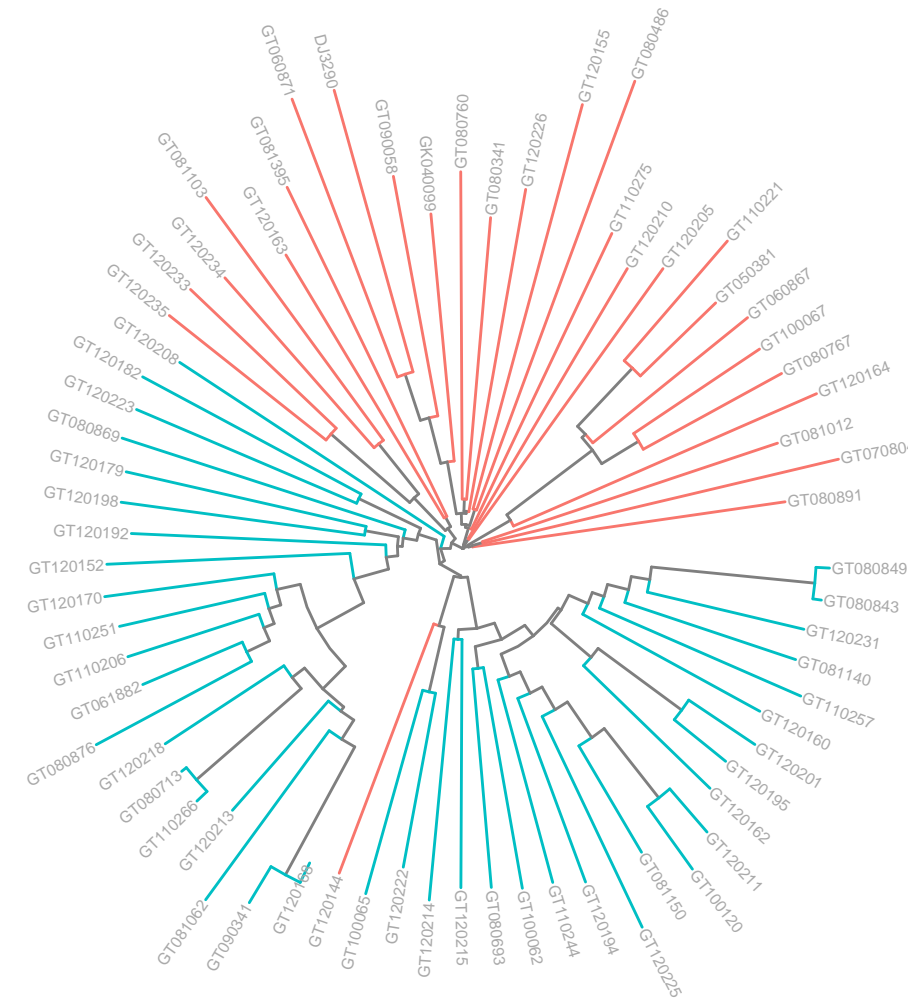**B**

Group

- 1
- 2
- NA

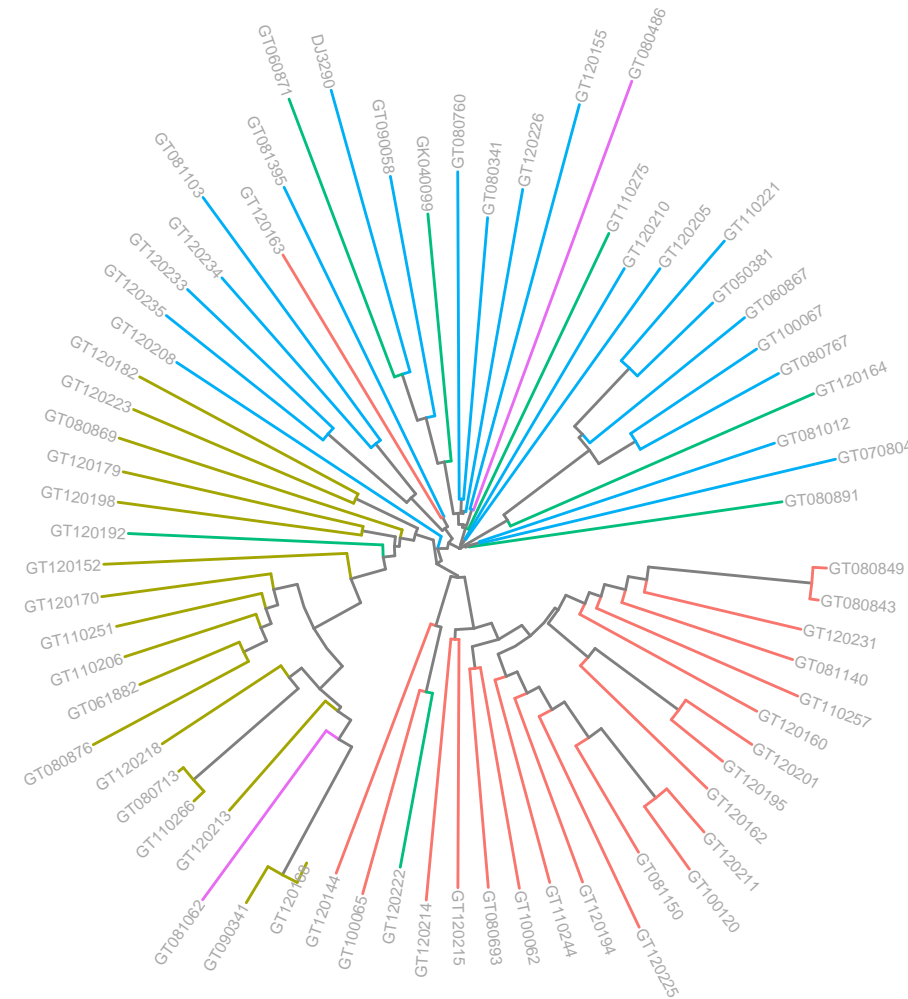**C**

Group

- 1
- 2
- 3
- 4
- 5
- NA

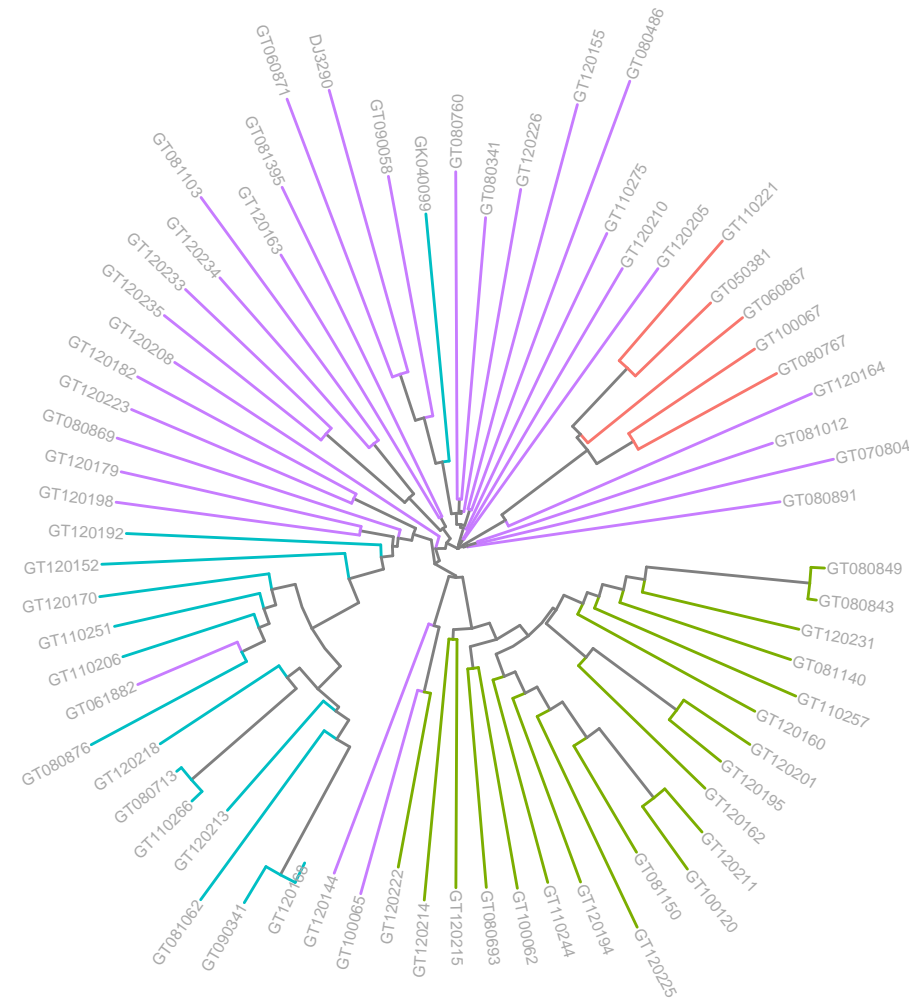

Group

- 1
- 2
- 3
- 4
- NA

Supplement: Supplementary file 7 — Additional file 7: Phylogenetic trees, generated in TASSEL using the Neighbour Joining method, to demonstrate the substructure present within the phenotyped panel. A) K = 2, the highest level of structure seen within the population following analysis with the relaxed SNP set, B) K = 5, the substructure present within the population following analysis with the relaxed SNP set. C) K = 4, the result following population structure analysis on the stringent SNP set. [file 12864_2021_7805_MOESM7_ESM.pdf]

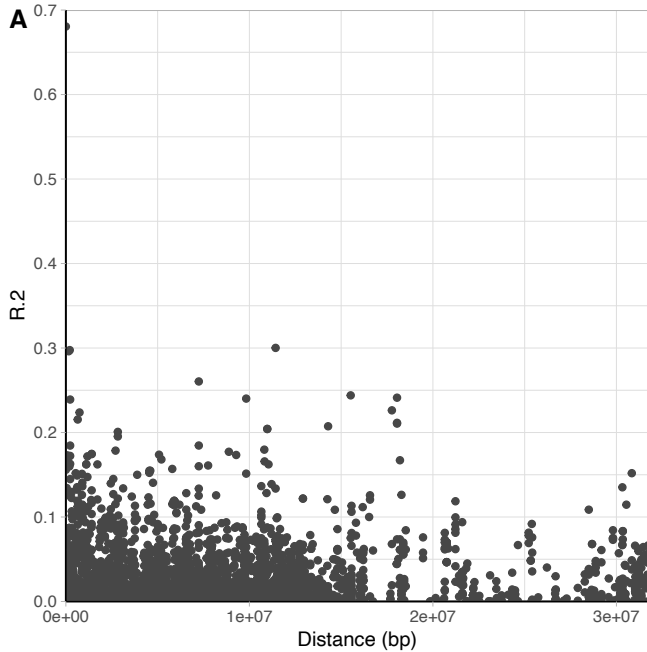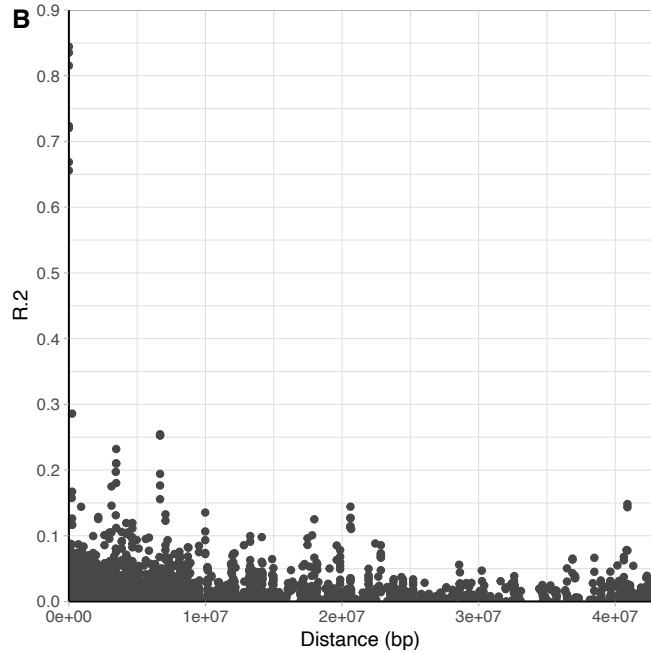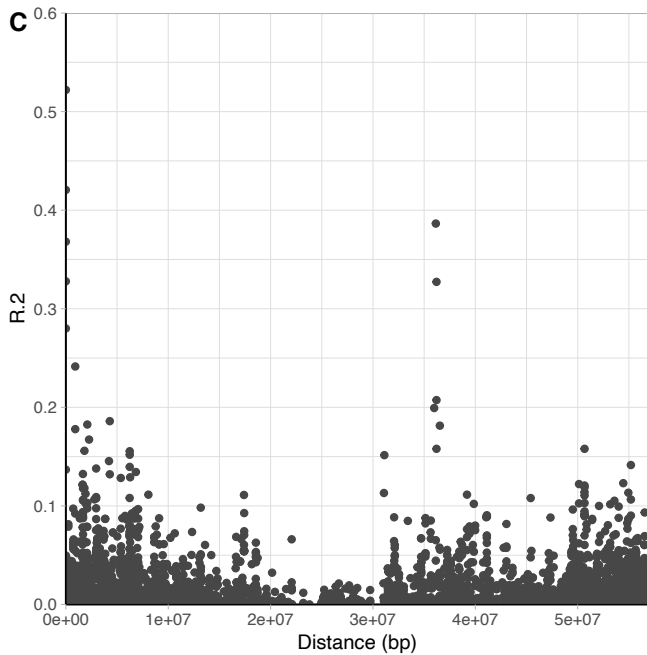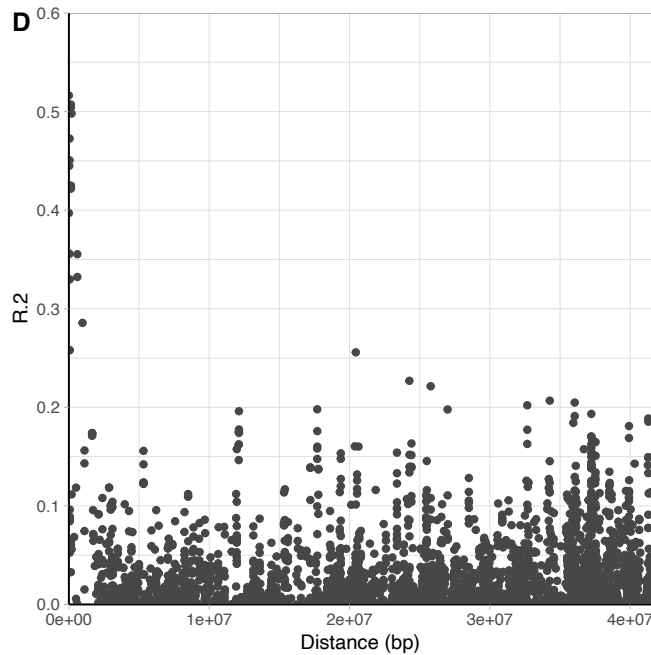

Supplement: Supplementary file 9 — Additional file 9: Linkage disequilibrium decay. A) Bo8g089990.1:453:T, miR172D candidate. B) Bo9g179000.1:2589:G, ELF6 candidate. C) Bo7g026810.1:124:G, FIP1 candidate. D) Bo7g104810.1:204:T, miR172D candidate. [file 12864_2021_7805_MOESM9_ESM.pdf]

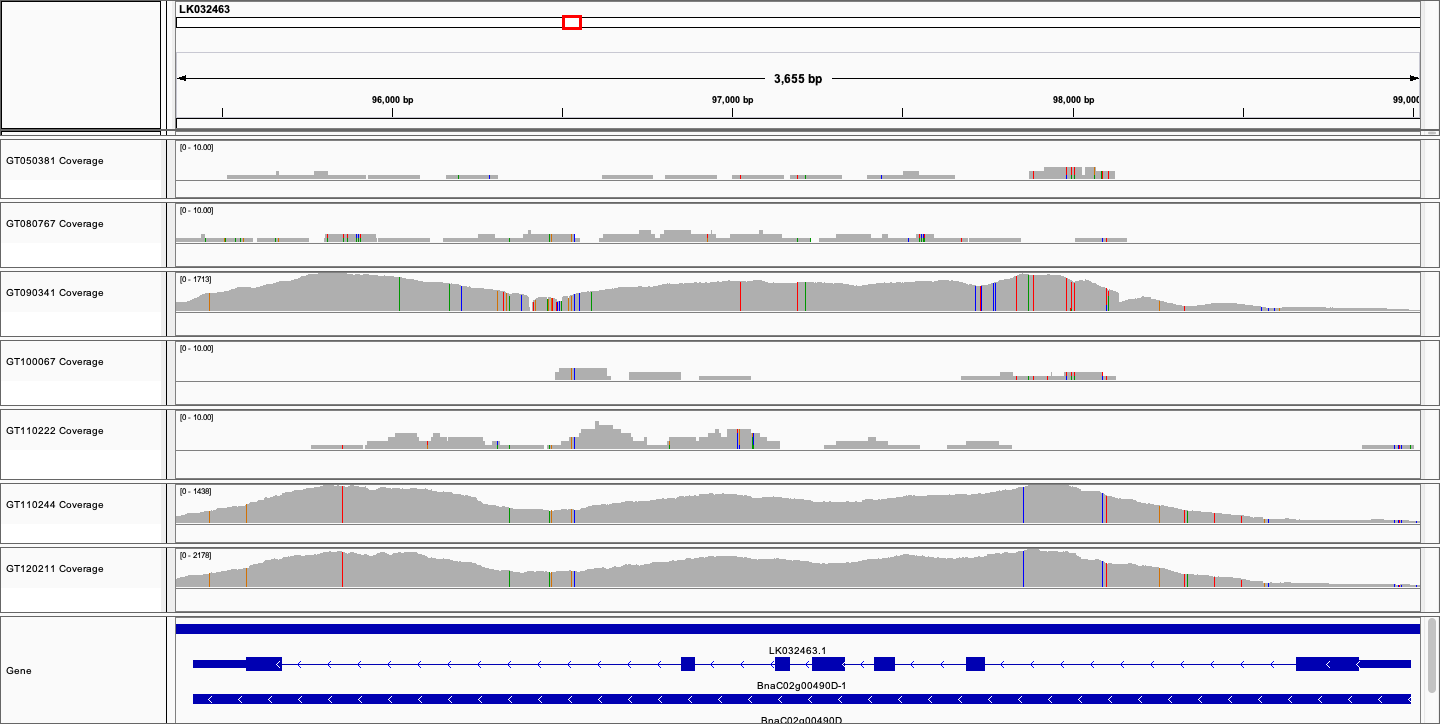

Supplement: Supplementary file 10 — Additional file 10: Mapping BoFLC.C2 using Darmor-bzh as a reference. Four rapid cycling accessions and three representative accessions for the rest of the population. [file 12864_2021_7805_MOESM10_ESM.png]
