# Supplementary material for: Validation of a novel associative transcriptomics pipeline in Brassica oleracea: identifying candidates for vernalisation response
Source: BMC Genomics. 2021 Jul 13;22:539. doi: 10.1186/s12864-021-07805-w (PMC8278714; doi:10.1186/s12864-021-07805-w)
Supplement: Supplementary file 8 — Additional file 8: Quantile-Quantile Plots for SNP associations with A) the DTF under NV conditions. GLM, with Q matrix correction for population structure B) the DTB after six-week pre-growth and 10ºC vernalisation for twelve-weeks. GLM, with Q matrix correction for population structure C) The difference in DTB following 5 ºC and 15 ºC vernalisation for six-weeks, after exposure to a ten-week pre-growth. GLM with Q matrix correction for population structure D) The DTF after exposure to six-week pre-growth, twelve weeks vernalisation 10 ºC. GLM with PCA correction for population structure. [file 12864_2021_7805_MOESM8_ESM.pdf]

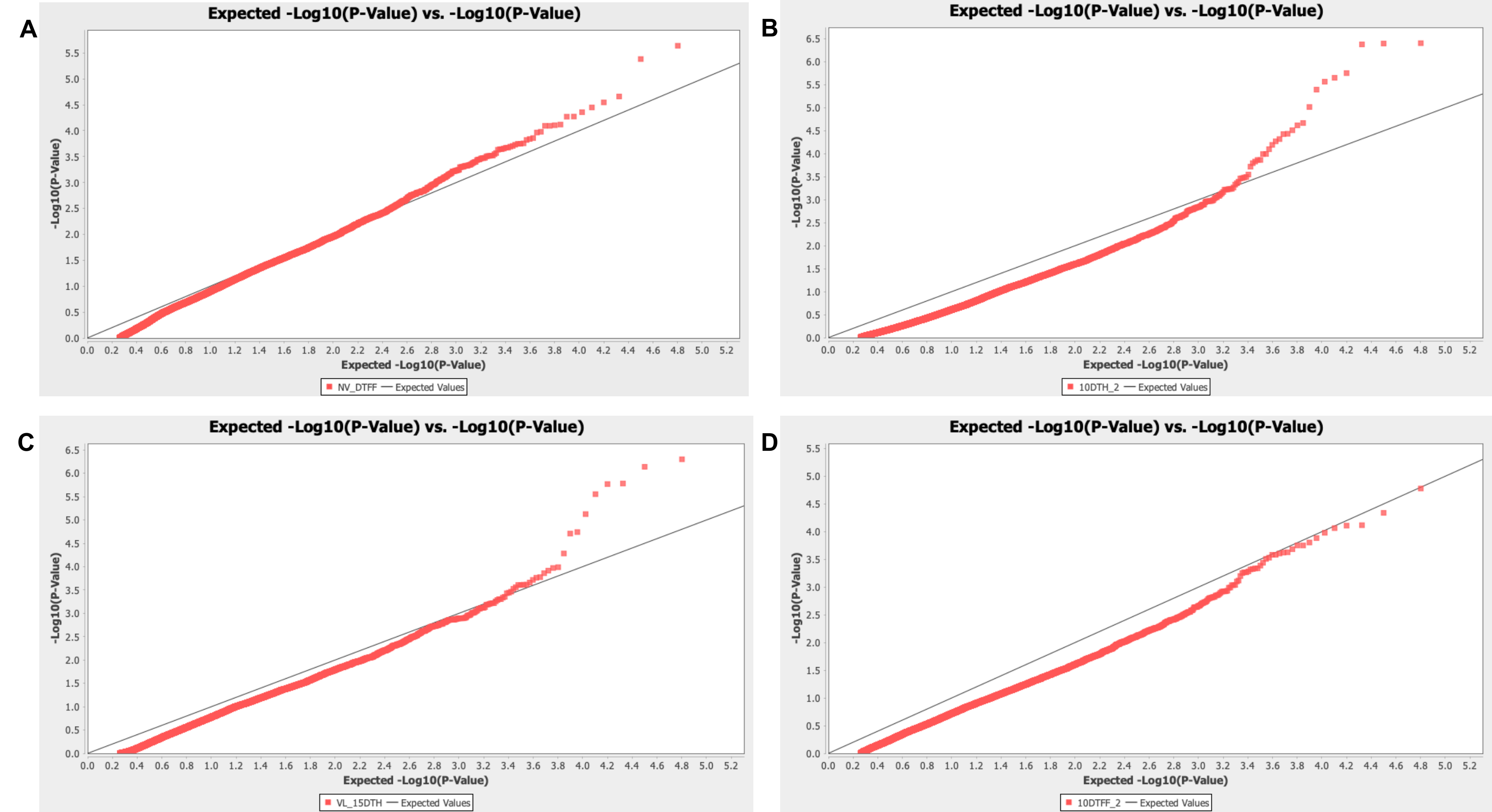
A
Expected -Log10(P-Value) vs. -Log10(P-Value)
-Log10(P-Value)
Expected -Log10(P-Value)
NV_DTFF — Expected Values
B
Expected -Log10(P-Value) vs. -Log10(P-Value)
-Log10(P-Value)
Expected -Log10(P-Value)
10DTH_2 — Expected Values
C
Expected -Log10(P-Value) vs. -Log10(P-Value)
-Log10(P-Value)
Expected -Log10(P-Value)
VL_15DTH — Expected Values
D
Expected -Log10(P-Value) vs. -Log10(P-Value)
-Log10(P-Value)
Expected -Log10(P-Value)
10DTFF_2 — Expected Values
